# The effects of 50 Hz magnetic field–exposed cell culture medium on cellular functions in FL cells

**DOI:** 10.1093/jrr/rrz020

**Published:** 2019-05-21

**Authors:** Yue Fei, Liling Su, Haifeng Lou, Chuning Zhao, Yiqin Wang, Guangdi Chen

**Affiliations:** 1 Bioelectromagnetics Laboratory, and Department of Reproductive Endocrinology of Women’s Hospital, Zhejiang University School of Medicine, 866 Yuhangtang Road, Hangzhou, China; 2 Department of Clinical Medicine, Jiangxi Medical College, Zhimin Road, Shangrao, China; 3 State Key Laboratory of Modern Optical Instrumentation (Zhejiang University), Centre for Optical and Electromagnetics Research, Zhejiang Provincial Key Laboratory for Sensing Technologies, JORCEP (Sino-Swedish Joint Research Center of Photonics), Zhejiang University, 866 Yuhangtang Road, Hangzhou, China

**Keywords:** relative permittivity, 50 Hz MF, culture medium, biological effects

## Abstract

Although extremely low frequency magnetic fields (ELF-MFs) have been classified as a possible carcinogen for humans by the International Agency for Research on Cancer (IARC), their biological effects and underlying mechanisms are still unclear. Our previous study indicated that ELF-MF exposure influenced the relative permittivity of the saline solution, suggesting that the MF exposure altered physical properties of the solution. To explore the biophysical mechanism of ELF-MF–induced biological effects, this study examined the effects of 50 Hz sinusoidal MF at 0–4.0 mT on the permittivity of culture medium with phase-interrogation surface plasmon resonance (SPR) sensing. Then, the biological effects of MF pre-exposed culture medium on cell viability, the mitogen-activated protein kinase (MAPK) signaling pathways, oxidative stress, and genetic stabilities were analyzed using Cell Counting Kit-8, western blot, flow cytometry, γH2AX foci formation, and comet assay. The results showed that SPR signals were decreased under MF exposure in a time- and dose-dependent manner, and the decreased SPR signals were reversible when the exposure was drawn off. However, MF pre-exposed culture medium did not significantly change cell viability, intracellular reactive oxygen species level, activation of the MARK signaling pathways, or genetic stabilities in human amniotic epithelial cells (FL cells). In conclusion, our data suggest that the relative permittivity of culture medium was influenced by 50 Hz MF exposure, but this change did not affect the biological processes in FL cells.

## INTRODUCTION

Increasing extremely low frequency magnetic field (ELF-MF) exposure, as generated by power lines and household electronic products, has raised public concern about potential adverse effects on human health. Epidemiological studies have suggested that ELF-MF exposure increased the risk of several neoplastic malignancies, including childhood leukemia, brain cancer and breast cancer [1–6]. Based on limited epidemiological evidences, in 2002, the International Agency for Research on Cancer (IARC) classified ELF-MF as a possible carcinogen for humans [7]. In addition, some studies have indicated that ELF-MFs could contribute to the etiology of neurodegenerative disorders, in particular of Alzheimer’s disease (AD) and amyotrophic lateral sclerosis (ALS) [8].

Regarding laboratory investigations, previous *in vivo* studies have examined the effects of chronic or acute ELF-MF exposure on the nervous system and behaviour, cardiovascular system responses, reproduction and development, and genotoxicity and cancer in laboratory animals [9–15]. A number of *in vitro* studies have been conducted to investigate the biological effects of ELF-MFs on numerous cell types, including possible effects on cell differentiation, cell proliferation, gene/protein expression, and genomic instability [16–20]. Unfortunately, the overall results concerning biological effects induced by ELF-MFs are contradictory and inconclusive, due to the previous studies over recent decades having different biological models, diverse exposure systems and exposure parameters, and different end points.

It is generally accepted that the energy generated by ELF-MFs is not sufficient to directly break molecular bonds, and a biophysical mechanism for low-energy ELF-MFs inducing biological effects remains to be elucidated. One of the considered mechanisms is that the interaction between the ELF-MF and the biological systems directly implies the involvement of oxidative stress, in particular by the radical pair mechanism, because the equilibrium of the elementary reaction producing a pair of radicals may be altered by a magnetic field. Thus, ELF-MFs may prolong the lifetime of free radicals and increase their concentration in living cells [21].

On the other hand, some studies have proposed biophysical and biochemical mechanisms for EMFs inducing biological effects, e.g. the MF influences the physicochemical properties of aqueous solutions, which in turn may modulate biological responses [22–24]. It has been reported that magnetic pretreatment of water altered cell density, size, and nuclear diameter of catfish hepatocytes [25], suggesting that physicochemical changes in the aqueous solution itself may mediate some MF bioeffects. Our previous study indicated that ELF-MF exposure influenced the relative permittivity of the saline solution [26]. The permittivity is a vital dielectric property of living cells or tissues, which is related to its biological activities [27]. It is reported that the relative permittivity differs between normal and cancer cells from different tissue types, and this forms the basis for using dielectric properties in diagnostic medicine [28, 29]. This observation led us to hypothesize that ELF-MFs may indirectly induce a change in the physical properties of culture medium, and thus affect the cellular functions.

To test this hypothesis, we first examined the effects of a 50 Hz sinusoidal MF on the permittivity of culture medium with phase-interrogation surface plasmon resonance (SPR) sensing. Then, the effects of 50 Hz MF pre-exposed culture medium on cellular viability, oxidative stress, the MAPK signaling pathways, and genetic stabilities in human amniotic epithelial cells (FL cells) were analyzed. The purpose of this study was to explore the biophysical mechanism for ELF-MF–induced cellular effects *in vitro.*

## MATERIAL AND METHODS

### The SPR sensing system

**Fig. 1.**
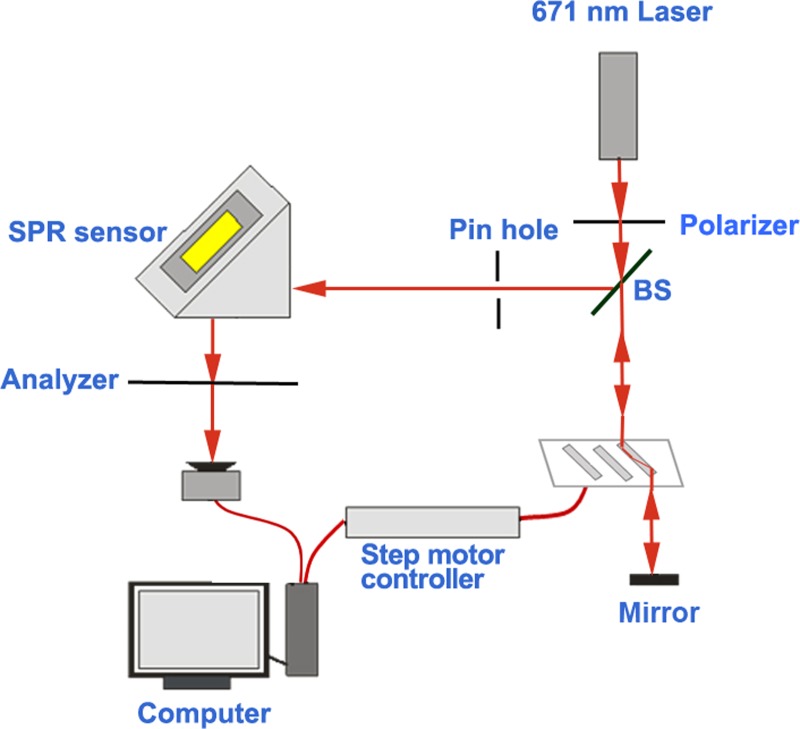
Schematic of the optical path SPR system.

The phase-sensitive SPR sensing system used in our study has been described previously [26]. The system included the light source, parallel-moving prism phase modulator (PM-PPM), SPR sensor and a photo detector. Figure 1 shows the schematic of our SPR sensing system. The light emitted from a stabilized laser with a wavelength of 671 nm went through a linear polarizer and a beam splitter, and was then incident into the PM-PPM equipped with three BK7 rhombic prisms. The light was reflected back into the prism by a mirror, filtered with a pin hole (to reduce the stray light), and struck the SPR sensor at an appropriate angle to reach the resonance condition. The reflected light from the SPR sensor passed through an analyzer, and then the interference light was detected by a photo detector. The SPR sensor in the Kretschmann configuration included a homemade microfluidic chip (size: 25.4 × 25.4 mm^2^) with one reaction chamber and one gold spot, and a right angle coupling prism.

### ELF-MF exposure system

The ELF-MF exposure system (OX9-1) used in this study was designed and provided by Dr Bao [30]. The exposure system included a C-shaped electromagnetic core, an excitation coil, a current amplifier, a field-strength controller, and a signal generator. The cell dishes or solution tubes were placed in the slot of a C-shaped electromagnet core on the cell hold platform to form a magnetic circuit. The excitation coil wrapped around the electromagnetic core was used to generate magnetic field. The excitation current for the excitation coil was provided by the current amplifier, and controlled by the field strength controller. The current amplifier can generate a 0–1 A current and input it to the excitation coil to generate a 0.01–25.37 mT magnetic field with a frequency between 0 and 340 Hz. The signal generator generates a frequency-adjustable signal for controlling the excitation current frequency. The linear relationship between electric voltage (mV) and magnetic intensity (mT) can be deduced as *y* = 13.44*x* + 0. 3371 (*R*^2^ = 0.9996), where *x* and *y* represent magnetic intensity and electric voltage, respectively. The magnetic intensities used in this study were calculated and adjusted by varying the electric voltage, e.g. 4.0 mT of MF can be offered by 54.10 mV of electric voltage.

### Exposure protocol

The cell culture medium (α-MEM medium with 10% FBS) was exposed to different exposure intensities (0–4.0 mT) of a 50 Hz sinusoidal MF for time periods (0–1 h), then the relative permittivity of the culture medium was measured by an SPR sensing system. For cell function analysis, the cells were exposed to the culture medium pre-exposed to a 50 Hz MF (4.0 mT for 1 h) for various time periods, e.g. the cells were cultured with pre-exposed medium for up to 48 h to detect cell viability.

### Cell culture

The FL cells were obtained from Shanghai Cell Bank of the Chinese Academy of Sciences and cultured in α-Minimum Essential Medium (HyClone, Thermo Scientific, Shanghai, China) supplemented with 10% heat-inactivated fetal bovine serum (HyClone) at 37°C in a humidified atmosphere containing 5% CO_2_.

### Cell viability analysis

The cell viability was determined using a Cell Counting Kit-8 (CCK-8, Dojindo Molecular Technologies, Inc., Kumamoto, Japan). The cells were seeded into 96-well plates at 5000 cells/well 12 h before exposure. The culture medium was pre-exposed to a 50 Hz MF for 1 h, then the cells were incubated with MF pre-exposed medium for an additional 12 h, 24 h, 36 h and 48 h at 37°C. CCK-8 reagent (10 μl per well) was added, and the cells were incubated for an additional 3 h at 37°C. The optical density value of each well was measured using a microplate reader (Varioskan Flash, Thermo Scientific, Waltham, MA, USA) at wavelength of 450 nm. As a positive control, the cells were treated with 100 μM H_2_O_2_ to inhibit viability. Each experiment was repeated three times.

### Intracellular reactive oxygen species detection

The intracellular reactive oxygen species (ROS) level was measured by flow cytometry using 2′, 7′-dichlorofluorescin diacetate (DCFH-DA, Beyotime Biotechnology). After exposure, the cells were washed with pre-warmed serum-free medium and incubated with DCFH-DA at a final concentration of 5 μM for 20 min in the dark. After washing three times to remove the extracellular DCFH-DA, the cells were detached with 0.25% trypsin-EDTA, and the fluorescence intensity was measured by flow cytometry. For each sample, 10 000 cells were measured. As a positive control, the cells were treated with 1.0 mM H_2_O_2_ for 1 h to induce cellular ROS. Each experiment was repeated six times.

### Immunofluorescent detection of γH2AX

Immunofluorescence staining was carried out as previously described [31, 32]. Primary mouse anti-γH2AX antibody was used (Millipore, Temecula, CA; 1:1000). Alexa Fluor 549–labelled goat anti-mouse secondary antibody (Zhongshan Goldenbridge Biotechnology, Beijing, China; 1:300) was used for visualization. Cell nuclei were stained with 4', 6-diamidino-2-phenylindole (DAPI, Sigma, Milwaukee, WI). Imaging of **γ**H2AX foci was performed using a Nikon fluorescence microscope (Nikon, Tokyo, Japan) with a ×40 oil objective. At least 200 cells were scored randomly from 5 to 10 fields. The number of γH2AX foci per cell and the percentage of γH2AX foci–positive cells were used as the indicators of DNA double-strand breaks (DSBs). Each experiment was independently repeated three times.

### Alkaline comet assay

Cellular DNA fragments were detected by the comet assay as described previously in detail [31]. Briefly, immediately after exposure, the cells were harvested, mixed with pre-warmed (37°C) 0.65% low-melting agarose and loaded onto slides pre-coated with 0.65% normal-melting agarose. The cells were then denatured with ice-cold lysis buffer [2.5 M NaCl, 1% sodium N-lauroyl sarcosinate, 0.1 M disodium EDTA, 10 mM Tris-HCl, pH 10.0] containing 1% Triton X-100 for 1 h, followed by treatment with 0.5 mg/ml DNase-free proteinase K (Amresco, OH, USA) in lysis buffer without Triton X-100 for 2 h at 37°C. The slices were immersed in the electrophoresis solution (0.3 M NaOH, 0.1% 8-hydroxyquinoline, 2% dimethylsulfoxide, 10 mM tetrasodium EDTA, pH 13) for 20 min to unwind the DNA, and then electrophoresed at a constant current of 300 mA (20 V, 0.4 V/m) for 20 min. After electrophoresis, the slices were neutralized in Tris buffer (0.4 M, pH 7.5) for 5 min and stained with Gel-Red (Biotium, Hayward, CA, USA). The DNA ‘comets’ were visualized under a fluorescence microscope (Nikon, Tokyo, Japan) with a ×20 objective. Approximately 200 comets were photographed for each independent exposure experiment, and the DNA damage parameters were analyzed using CASP 1.2.2 software (Krzysztof Konca, Wroclaw, Poland). The percentage of tail DNA and Olive tail moment for each comet were calculated as indicators of DNA fragments. Each experiment was independently repeated three times.

### Western blot analysis

Western blotting was performed as previously described [33]. Total proteins were extracted from cells after lysing with lysis buffer, and quantified using a bicinchoninic acid (BCA) protein assay kit (Beyotime). The equal amounts of total proteins were separated by sodium dodecyl sulfate polyacrylamide gel electrophoresis and transferred to a nitrocellulose membrane (Whatman, Freiburg, Germany). The membrane was blocked with 3% BSA in Tris-buffered saline and Tween 20 (TBST) buffer for 2 h, and blotted with the corresponding primary antibody at 4°C overnight followed by incubation with IR-Dye conjugated secondary antibody goat anti-mouse or goat anti-rabbit (Li-COR Biosciences, B80908–02 or B81009–02, 1:10 000) for 1 h. Finally, the blot was visualized using an Odyssey infrared imaging system (Li-COR Biosciences) and then quantified using densitometry. The primary mouse anti-Actin (sc47778, 1:2000) antibodies were from Sigma (St Louis, MO, USA); the rabbit anti-p38 MAPK (8690, 1:1000), anti-p-p38 MAPK (4511, 1:1000), anti-ERK and anti-p-ERK (4370, 1:1000) antibodies were from Cell Signaling Technology (CST, MA, USA).

## RESULTS

### The effects of 50 Hz MF exposure on SPR signals of culture medium

**Fig. 2.**
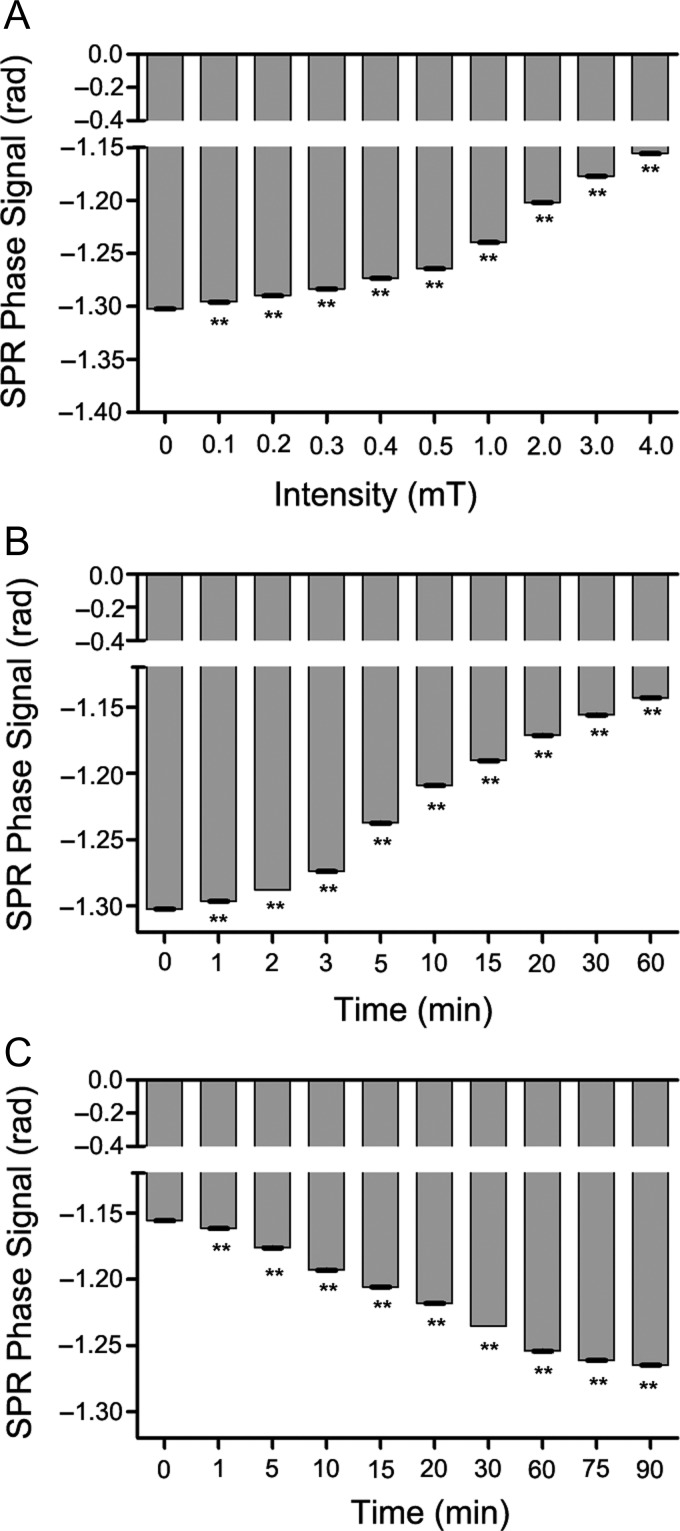
The effects of a 50 Hz MF exposure on SPR signals of culture medium. (A) MF exposure decreased the SPR signal in a dose-dependent manner. (B) MF exposure decreased the SPR signal in a time-dependent manner. (C) Recovery of SPR signals of culture medium when the MF exposure was drawn off. SPR signal (that has been decreased by MF earlier) increased (i.e. recovered) in a time-dependent way. Values represent mean ± SD from three independent experiments. **P < 0.01, compared with the sham group.

To examine the effects of a 50 Hz MF on the relative permittivity of culture medium, we first measured the SPR signal of α-MEM medium with 10% FBS (complete medium) under different exposure intensities of ELF-MF for 30 min. The results showed that the MF decreased the SPR signal in a dose-dependent manner, and that a 50 Hz MF as low as 0.1 mT could significantly decrease the SPR signal (Fig. 2A). The complete medium was exposed to 4.0 mT MF for different exposure time periods and the SPR signals of the medium were measured. The SPR signal was immediately decreased after MF exposure for 1 min, and continuously decreased in a time-dependent manner (Fig. 2B). In addition, we evaluated the recovery of SPR signals of complete medium when MF exposure was drawn off, and found that the MF-decreased SPR signals began to rise immediately, and gradually increased during the recovery time, suggesting that the permittivity decrease caused by a MF is reversible (Fig. 2C). Taken together, these results demonstrated that MF exposure reversibly decreased the permittivity of the complete medium in a time- and dose-dependent way.

### The effects of a 50 Hz MF pre-exposed culture medium on cellular functions in FL cells

**Fig. 3.**
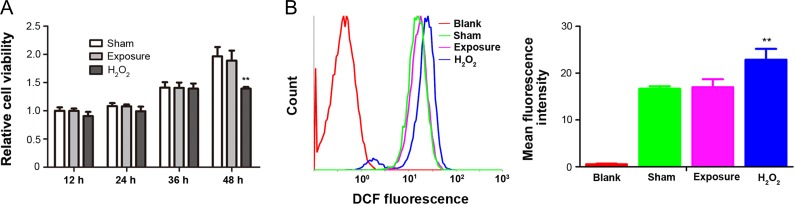
The effects of treatment with culture medium pre-exposed to a 50 Hz MF on cellular viability and ROS production in FL cells. The culture medium was pre-exposed by a 50 Hz MF for 1 h, then FL cells were incubated with sham (sham-treated) or 50 Hz MF pre-exposed culture medium (exposure-treated) for various durations. (A) Relative cell viabilities were examined by CCK8. (B) ROS levels were evaluated by flow cytometry using DCFH-DA. Left, representative flow cytometry histograms of DCF fluorescence of the sham-treated cells (green line), exposure-treated cells (pink line), H_2_0_2_-treated cells (blue line), and the blank control cells not probed with DCFH-DA (red line); right, quantitative analysis of DCF mean fluorescence intensity (MFI). Values represent mean ± SD from six independent experiments. **P < 0.01, compared with the sham group.

Since the permittivity of medium was decreased by 50 Hz MF exposure, we were interested in whether this change in permittivity could affect the cellular functions. Therefore, FL cells were chosen as a model to determine whether MF pre-exposed culture medium led to aberrant cell viability, oxidative stress (i.e. intracellular ROS level), MARK signaling pathways, and genetic instabilities. First, culture medium pre-exposed to a MF at 4.0 mT for 1 h (conditioned medium) was used to culture cells for various time periods, and the cell viability and oxidative stress were examined. The results showed that incubation with conditioned medium for 12–48 h did not significantly inhibit cell viability (Fig. 3A). Oxidative stress refers to elevated intracellular levels of ROS under various cellular stresses that cause damage to lipids, proteins and DNA. As shown in Fig. 3B, incubation with conditioned medium for 1 h did not significantly induce ROS production in FL cells.

**Fig. 4.**
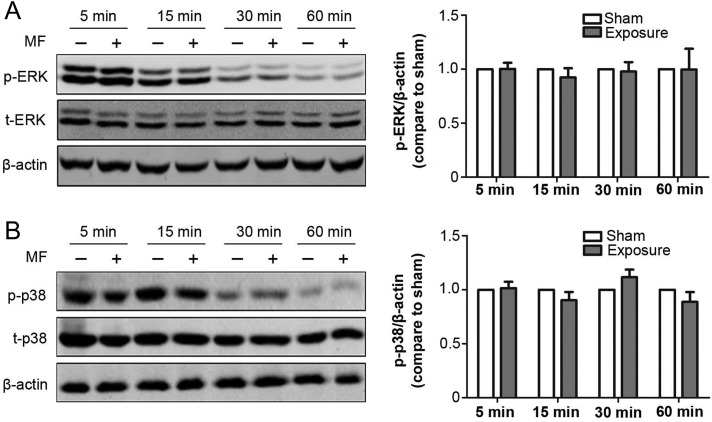
The effects of treatment with culture medium pre-exposed to a 50 Hz MF on expression levels of proteins in the MAPK signaling pathways in FL cells. The culture medium was pre-exposed to a 50 Hz MF for 1 h, then FL cells were incubated with sham or MF pre-exposed culture medium for 1 h. Left panel, representative western blot images show the ERK and p-ERK (A), p38 and p-p38 (B) levels in FL cells after incubation with the culture medium pre-exposed to a 50 Hz MF for various durations as indicated. Right panel, histograms showing the quantitative analysis of protein bands of western blot analysis. Values represent mean ± SD from six independent experiments.

Next, we investigated the effect of treatment with conditioned medium on the expression level of proteins involved in MAPK (i.e. ERK and p38) signaling pathways in FL cells, which regulate many fundamental cellular functions such as cell proliferation, growth, and survival. As shown in Fig. 4, ERK and p38 phosphorylation and expression level did not differ from that in the sham-exposed group after exposure for 5 min to 60 min.

**Fig. 5.**
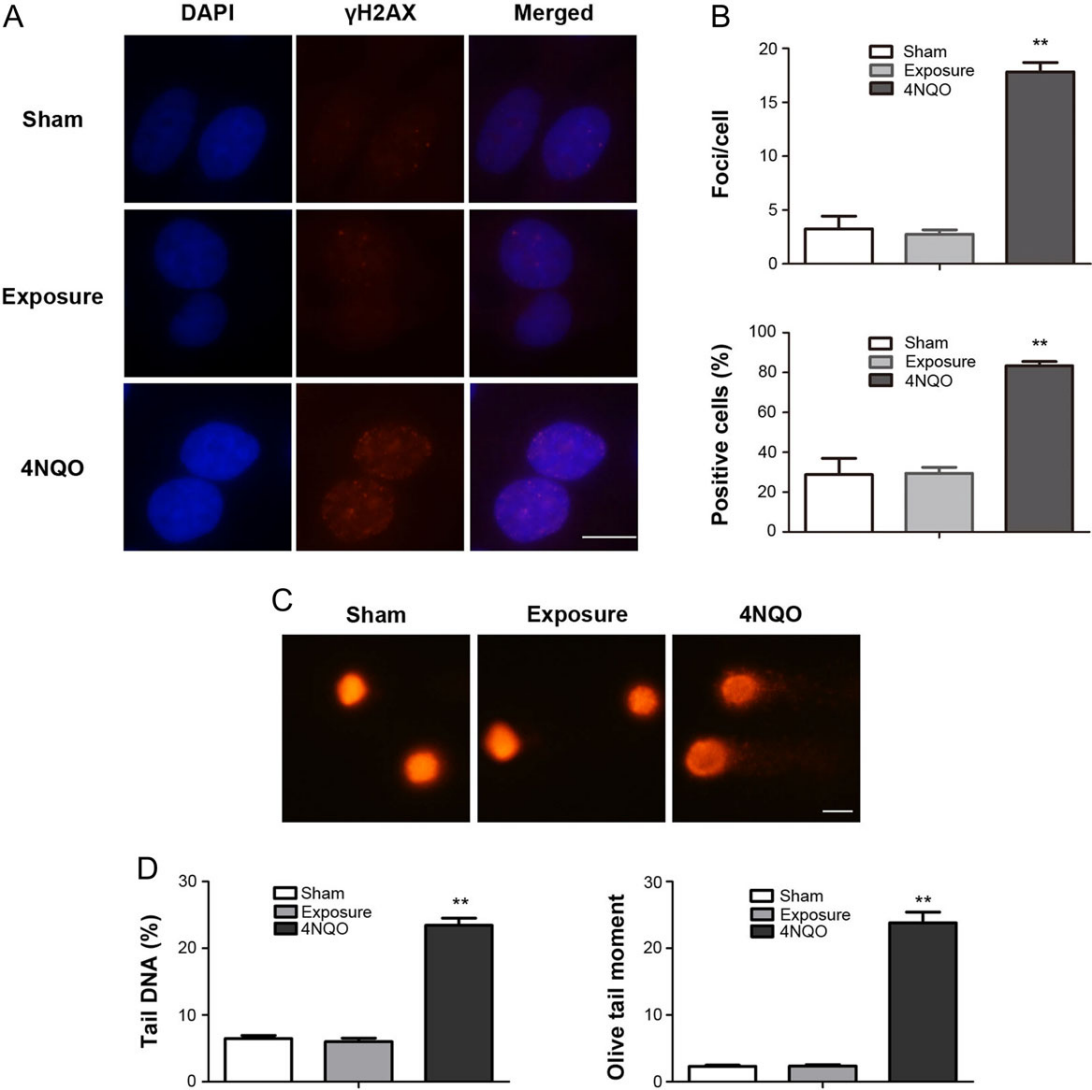
The effects of treatment with culture medium pre-exposed to a 50 Hz MF on cellular DNA in FL cells. The culture medium was pre-exposed to a 50 Hz MF for 1 h, then FL cells were incubated with sham or MF pre-exposed culture medium for 1 h. (A) Representative images of γH2AX immunofluorescent staining of FL cells. Red dots indicate γH2AX foci; nuclei are stained blue with DAPI. (B) Histograms showing the average numbers of γH2AX foci per cell (upper) and the percentage of γH2AX foci–positive cells (lower). (C) Representative alkaline comet images showing DNA fragmentation in FL cells. (D) Histograms showing the data for tail DNA (%) (left), and Olive tail moment (right) in FL cells. Values represent mean ± SD from three independent experiments. **P < 0.01, compared with the sham group. Scale bar, 10 μm.

Further, to reveal the influence of the change in permittivity on cellular DNA, we examined the DNA DSB and DNA fragmentation status after treatment with conditioned medium using γH2AX foci formation and the comet assay. The results showed no significant differences in average foci per cell or percentage of positive cells, suggesting treatment with conditioned medium did not induce DNA DSBs in FL cells (Fig. 5A and B). The comet assay also revealed that treatment with conditioned medium did not significantly change the percentage of tail DNA or Olive tail moment (Fig. 5C and D).

Overall, these results indicated that treatment with a 50 Hz MF pre-exposed culture medium did not result in obvious aberrant cellular functions in FL cells.

## DISCUSSION

In this study, we measured the relative permittivity of culture medium under a 50 Hz MF based on an SPR system, and found that SPR signals were decreased under MF exposure in a time- and dose-dependent manner. In addition, we found that the decreased SPR signals were reversible when the exposure was drawn off. Furthermore, we found treatment with MF pre-exposed culture medium did not result in obvious aberrant cellular functions in FL cells. To the best of our knowledge, this study for the first time examined the relative permittivity of culture medium under a 50 Hz MF with the help of the SPR system and explored a new potential biophysical mechanism for a 50 Hz MF acting on cells.

Consistent with our previous study that MF exposure induced changes in the relative permittivity of a saline solution, this study also suggest that the relative permittivity of culture medium was influenced by MF exposure. Previous studies reported that MF could change the physicochemical properties of water, e.g. electric conductivity, surface-tension force, refraction index, dielectric constant, and evaporation amount [34–40], and magnetized water could effectively influence the oxidant–antioxidant balance, e.g. decrease the amount of malondialdehyde, and increase the superoxide dismutase activity and intracellular GSH levels [41, 42]. However, our study found that the change in the relative permittivity of the culture medium induced by the MF did not influence the cellular functions. It should be noted that the sensitivity and resolution of our SPR system is ~1900 rad/RIU and 3.421 × 10^−7^ RIU, which could detect a very tiny change in the SPR phase signal. In fact, the significant change in the SPR phase signal detected in this study may indicate a very small alteration to the relative permittivity of the culture medium. Thus, the biological effects of treatment with culture medium pre-exposed to a 50 Hz MF on FL cells may be very weak, and thus could not be detected by the techniques used in this study. Our data indicated that the permittivity of the culture medium that was decreased by the MF was reversible when MF exposure was drawn off, suggesting the effects of the pre-exposed culture medium on cellular functions would have been short-lived. These may be the reasons for the negative results in our study. Future studies should be conducted to evaluate the permittivity of the culture medium under real-time MF exposure conditions, and the real-time cellular effects induced by MF-exposed culture medium.

In conclusion, our data suggested that MF exposure decreased the relative permittivity of culture medium in a time- and dose-dependent manner; however, this change of culture medium did not result in obviously aberrant cellular functions in FL cells.
